# Supplementary material for: Assessing the performance of genome-wide association studies for predicting disease risk
Source: PLoS One. 2019 Dec 5;14(12):e0220215. doi: 10.1371/journal.pone.0220215 (PMC6894795; doi:10.1371/journal.pone.0220215)
Supplement: S1 Table — (PDF) [file pone.0220215.s001.pdf]

S1 Table

GWAS-ROCS ID	Condition/Phenotype	Pawitan et al. Heritability
GR00001	Abdominal aortic aneurysm	0.004162347
GR00002	Abdominal aortic aneurysm	0.021762399
GR00003	Acne (severe)	0.042017796
GR00004	Acute lymphoblastic leukemia (childhood)	0.103370717
GR00005	Acute lymphoblastic leukemia (childhood)	0.087244098
GR00006	Acute lymphoblastic leukemia (childhood)	0.008476861
GR00007	Acute-on-chronic liver failure in hepatitis B	0.027527524
GR00008	Adolescent idiopathic scoliosis	0.00985719
GR00009	Adolescent idiopathic scoliosis	0.06022384
GR00010	Age-related macular degeneration	0.284701365
GR00011	Age-related macular degeneration	0.058090794
GR00012	Age-related macular degeneration	0.272397476
GR00013	Age-related macular degeneration	0.021835469
GR00014	Age-related macular degeneration	0.196292911
GR00015	Age-related macular degeneration	0.064377847
GR00016	Age-related macular degeneration	0.071985892
GR00017	Aggressive periodontitis	0.042284365
GR00018	Allergic disease	0.022049233
GR00019	Allergic sensitization	0.029839602
GR00020	Alzheimer's disease	0.008889895
GR00021	Alzheimer's disease	0.009514793
GR00022	Alzheimer's disease	0.004295769
GR00023	Alzheimer's disease	0.013183269
GR00024	Alzheimer's disease	0.059322564
GR00025	Alzheimer's disease	0.000999421
GR00026	Alzheimer's disease	0.013237706
GR00027	Alzheimer's disease	0.039542708
GR00028	Alzheimer's disease	0.041702778
GR00029	Alzheimer's disease	0.03240962
GR00030	Alzheimer's disease	0.063687882
GR00031	Alzheimer's disease	0.064521106
GR00032	Alzheimer's disease	0.047689508
GR00033	Amyotrophic lateral sclerosis	0.012234181
GR00034	Amyotrophic lateral sclerosis	0.045525479

GR00035	Amyotrophic lateral sclerosis	0.031089518
GR00036	Anorexia nervosa	0.019832795
GR00037	Anti-dsDNA status	0.067367823
GR00038	Anti-dsDNA status	0.204944933
GR00039	Anxiety disorder	0.003251616
GR00040	Aortic valve stenosis	0.014808642
GR00041	Arthritis (juvenile idiopathic)	0.009764383
GR00042	Arthritis (juvenile idiopathic)	0.03120038
GR00043	Asthma	0.023900759
GR00044	Asthma	0.03999514
GR00045	Asthma	0.019054731
GR00046	Asthma	0.003839564
GR00047	Asthma	0.005635514
GR00048	Asthma	0.009096351
GR00049	Asthma	0.035434514
GR00050	Atopic dermatitis	0.008563549
GR00051	Atopic dermatitis	0.005507953
GR00052	Atrial fibrillation	0.036307888
GR00053	Atrial fibrillation	0.127761296
GR00054	Atrial fibrillation	0.002327245
GR00055	Atrial fibrillation	0.074946298
GR00056	Autism	0.006734456
GR00057	Autism	0.014552163
GR00058	Autoimmune hepatitis type-1	0.100160246
GR00059	B cell non-Hodgkin lymphoma	0.013232706
GR00060	Barrett's esophagus	0.004907046
GR00061	Barrett's esophagus	0.024962713
GR00062	Basal cell carcinoma	0.007403442
GR00063	Basal cell carcinoma	0.014777768
GR00064	Behcet's disease	0.01485452
GR00065	Behcet's disease	0.00551565
GR00066	Behcet's disease	0.031025664
GR00067	Behcet's disease	0.026699934
GR00068	Bipolar disorder	0.045755291
GR00069	Bipolar disorder	0.013243625
GR00070	Black vs. non-black hair color	0.279026726
GR00071	Bladder cancer	0.003302355
GR00072	Bladder cancer	0.012165591

GR00073	Bladder cancer	0.002941046
GR00074	Bladder cancer	0.034383014
GR00075	Blond vs non-blond hair color	0.104755996
GR00076	Blond vs. brown hair color	0.07988577
GR00077	Blue vs. green eyes	0.012740387
GR00078	Breast cancer	0.038264559
GR00079	Breast cancer	0.018139919
GR00080	Breast cancer	0.006230443
GR00081	Breast cancer	0.00456076
GR00082	Breast cancer	0.024954645
GR00083	Breast cancer	0.008943937
GR00084	Breast cancer	0.029914467
GR00085	Breast cancer	0.017630407
GR00086	Breast cancer	0.009105047
GR00087	Breast cancer	0.003570821
GR00088	Breast cancer	0.00319403
GR00089	Breast cancer	0.003396067
GR00090	Breast cancer	0.02029287
GR00091	Breast cancer	0.002849713
GR00092	Breast cancer	0.048296101
GR00093	Breast cancer	0.021239842
GR00094	Breast cancer	0.023339868
GR00095	Breast cancer	0.056500656
GR00096	Breast cancer	0.024113253
GR00097	Breast cancer	0.004392492
GR00098	Breast cancer	0.05288057
GR00099	Breast cancer	0.034816052
GR00100	Breast cancer	0.01881074
GR00101	Breast cancer	0.00510482
GR00102	Bronchopulmonary dysplasia	0.086715224
GR00103	Brown vs. non-brown hair color	0.082153762
GR00104	Brugada syndrome	0.155369468
GR00105	Burning and freckling	0.073277624
GR00106	Calcific aortic valve stenosis	0.009144027
GR00107	Cardia gastric cancer	0.00524288
GR00108	Cardiac repolarization	0.024371607
GR00109	Cardiovascular disease risk factor	0.004395567
GR00110	Cardiovascular disease risk factor	0.005122735

GR00111	Cardiovascular disease risk factor	0.012372754
GR00112	Carotid intima media thickness, plaque	0.002347839
GR00113	Case-only systemic lupus erythematosus	0.085437469
GR00114	Celiac disease	0.331635197
GR00115	Cervical cancer	0.029466815
GR00116	Cervical cancer	0.010527487
GR00117	Cervical cancer	0.013311215
GR00118	Cholangiocarcinoma in primary sclerosing cholangitis	0.016054486
GR00119	Chronic bronchitis in chronic obstructive pulmonary disease	0.009217093
GR00120	Chronic hepatitis B infection	0.105951642
GR00121	Chronic hepatitis B infection	0.118375909
GR00122	Chronic hepatitis C infection	0.007928738
GR00123	Chronic kidney disease	0.002439075
GR00124	Chronic kidney disease	0.00500031
GR00125	Chronic kidney disease	0.004448058
GR00126	Chronic kidney disease	0.011845313
GR00127	Chronic kidney disease	0.014186527
GR00128	Chronic lymphocytic leukemia	0.107535678
GR00129	Chronic lymphocytic leukemia	0.02346047
GR00130	Chronic lymphocytic leukemia	0.034287341
GR00131	Chronic obstructive pulmonary disease	0.026073357
GR00132	Chronic obstructive pulmonary disease	0.023812372
GR00133	Chronic obstructive pulmonary disease	0.027806976
GR00134	Cleft lip	0.05404079
GR00135	Colon cancer	0.011488937
GR00136	Colorectal cancer	0.023427414
GR00137	Colorectal cancer	0.010684586
GR00138	Colorectal cancer	0.010839925
GR00139	Colorectal cancer	0.005617641
GR00140	Colorectal cancer	0.020321818
GR00141	Colorectal cancer	0.003561416
GR00142	Colorectal cancer	0.025715914
GR00143	Colorectal cancer	0.020198624
GR00144	Colorectal cancer	0.002971312
GR00145	Colorectal cancer	0.008986206
GR00146	Colorectal cancer	0.045252525
GR00147	Colorectal cancer	0.032525407

GR00148	Colorectal cancer	0.017507445
GR00149	Colorectal cancer	0.010676326
GR00150	Colorectal cancer	0.007081238
GR00151	Colorectal cancer	0.010869981
GR00152	Colorectal cancer	0.000415944
GR00153	Colorectal cancer	0.016858961
GR00154	Colorectal cancer	0.008055539
GR00155	Colorectal cancer	0.037323325
GR00156	Combined Crohn's disease and sarcoidosis	0.004861123
GR00157	Congenital heart malformation	0.01166589
GR00158	Corneal astigmatism	0.031325538
GR00159	Coronary artery disease	0.002338181
GR00160	Coronary artery disease	0.012541095
GR00161	Coronary artery disease	0.015861535
GR00162	Coronary artery disease	0.0324426
GR00163	Coronary artery disease	0.011692444
GR00164	Coronary artery disease	0.002917435
GR00165	Coronary artery disease	0.036175299
GR00166	Coronary artery disease	0.005936919
GR00167	Coronary artery disease	0.163105336
GR00168	Coronary artery disease	0.015613164
GR00169	Coronary artery disease	0.02912354
GR00170	Coronary artery disease	0.010232528
GR00171	Coronary artery disease	0.016804438
GR00172	Coronary artery disease	0.004162095
GR00173	Coronary artery disease	0.005884303
GR00174	Coronary artery disease	0.008804034
GR00175	Craniofacial microsomia	0.203447856
GR00176	Creutzfeldt-Jakob disease	0.018342418
GR00177	Crohn's disease	0.123648489
GR00178	Crohn's disease	0.043105554
GR00179	Crohn's disease	0.062283135
GR00180	Crohn's disease	0.091114789
GR00181	Crohn's disease	0.014560295
GR00182	Crohn's disease	0.149627553
GR00183	Curve progression in adolescent idiopathic scoliosis	0.020970229
GR00184	Dementia with Lewy bodies	0.082521523
GR00185	Diabetic nephropathy	0.042472652

GR00186	Diabetic nephropathy	0.004488554
GR00187	Diarrhoeal Disease	0.00956929
GR00188	Diarrhoeal Disease	0.008484939
GR00189	Diarrhoeal Disease	0.003612698
GR00190	Diarrhoeal Disease	0.006344985
GR00191	Diffuse large B cell lymphoma	0.032040324
GR00192	Digestive system disease	0.028155603
GR00193	Disc degeneration (lumbar)	0.004954629
GR00194	Disease-free survival in breast cancer	0.119442865
GR00195	Drinking behavior	0.105737067
GR00196	Drug abuse	0.015247863
GR00197	Dupuytren's disease	0.12598796
GR00198	Dupuytren's disease	0.168165597
GR00199	Early onset inflammatory bowel disease	0.011425058
GR00200	Early-onset obesity	0.041513415
GR00201	End-stage renal disease in Type 1 diabetics (Women)	0.02511986
GR00202	Endometrial cancer	0.004570587
GR00203	Endometrial cancer	0.019239604
GR00204	Endometrial cancer	0.019831763
GR00205	Endometriosis	0.012651903
GR00206	Endometriosis	0.022296848
GR00207	Endometriosis	0.017563298
GR00208	Enteric fever	0.067629
GR00209	Epilepsy	0.021662925
GR00210	Epilepsy	0.028071611
GR00211	Epilepsy	0.0162159
GR00212	Epilepsy	0.019940492
GR00213	Epithelial ovarian cancer	0.042557478
GR00214	Epstein Barr virus nuclear antigen 1 IgG seropositivity	0.001652333
GR00215	Esophageal cancer	0.033814518
GR00216	Esophageal cancer	0.029256097
GR00217	Esophageal cancer	0.078832239
GR00218	Esophageal cancer	0.026815559
GR00219	Esophageal cancer	0.016555094
GR00220	Esophageal cancer	0.013780689
GR00221	Esophageal cancer	0.006138652
GR00222	Ewing sarcoma	0.105463706

GR00223	Febrile seizures	0.074429178
GR00224	Febrile seizures	0.099680529
GR00225	Febrile seizures	0.026919385
GR00226	Follicular lymphoma	0.046508738
GR00227	Follicular lymphoma	0.028768778
GR00228	Follicular lymphoma	0.077641646
GR00229	Fractures (vertebral)	0.0148789
GR00230	Freckles	0.038379054
GR00231	Gallbladder cancer	0.033760555
GR00232	Gastric cancer	0.010868997
GR00233	Glaucoma	0.026029063
GR00234	Glaucoma	0.057532852
GR00235	Glaucoma	0.019826004
GR00236	Glaucoma	0.018097181
GR00237	Glaucoma	0.017521126
GR00238	Glioma	0.032164781
GR00239	Glioma	0.006910395
GR00240	Glioma	0.044742294
GR00241	Goiter	0.056663518
GR00242	Gout	0.049423696
GR00243	Gout	0.137279995
GR00244	Gout	0.140782472
GR00245	Graves disease	0.137838173
GR00246	Graves disease	0.119399435
GR00247	Graves disease	0.027304839
GR00248	Heart failure	0.166499615
GR00249	Heart failure	0.026016022
GR00250	Helicobacter pylori serologic status	0.022176897
GR00251	Hepatitis B	0.142326493
GR00252	Hepatitis B	0.037785111
GR00253	Hepatitis B	0.060586149
GR00254	Hepatitis B	0.081545902
GR00255	Hepatitis B	0.053396341
GR00256	Hepatitis C induced liver cirrhosis	0.05108722
GR00257	Hepatocellular carcinoma	0.014868406
GR00258	Hepatocellular carcinoma	0.029119573
GR00259	Hepatocellular carcinoma	0.023644701
GR00260	Hepatocellular carcinoma	0.013122643

GR00261	High serum lipase activity	0.035465512
GR00262	Hodgkin's lymphoma	0.054789842
GR00263	Hyperemesis gravidarum	0.019318486
GR00264	Hypertension	0.001824211
GR00265	Hypertension	0.008812926
GR00266	Hypertriglyceridemia	0.104046998
GR00267	Idiopathic pulmonary fibrosis	0.10318996
GR00268	Idiopathic pulmonary fibrosis	0.126468724
GR00269	Immunoglobulin light chain (AL) amyloidosis	0.075388818
GR00270	Infantile hypertrophic pyloric stenosis	0.065542662
GR00271	Infantile hypertrophic pyloric stenosis	0.051090279
GR00272	Inflammatory bowel disease	0.086864909
GR00273	Inflammatory bowel disease	0.103875054
GR00274	Inguinal hernia	0.006163864
GR00275	Insomnia (caffeine-induced)	0.11860224
GR00276	Interstitial lung disease	0.160940486
GR00277	Intracerebral hemorrhage	0.010643831
GR00278	Intracranial aneurysm	0.030923913
GR00279	Intracranial aneurysm	0.019683563
GR00280	Intracranial aneurysm	0.031309826
GR00281	Intracranial aneurysm	0.020466685
GR00282	Irritable bowel syndrome	0.01273299
GR00283	Ischemic stroke	0.008444172
GR00284	Ischemic stroke	0.003168836
GR00285	Ischemic stroke	0.001327427
GR00286	Ischemic stroke	0.005851086
GR00287	Ischemic stroke	0.003317405
GR00288	Ischemic stroke	0.024617484
GR00289	Kawasaki disease	0.09915466
GR00290	Kawasaki disease	0.016850814
GR00291	Kidney disease	0.002010004
GR00292	Kidney disease	0.019045123
GR00293	Kidney disease	0.011483542
GR00294	Kidney disease	0.005471561
GR00295	Kidney disease	0.005960596
GR00296	Kidney stones	0.004996789
GR00297	Knee osteoarthritis	0.012767989
GR00298	Knee osteoarthritis	0.010682656

GR00299	Late onset Alzheimer's disease	0.016893913
GR00300	Late onset Alzheimer's disease	0.143125219
GR00301	Leishmaniasis (visceral)	0.016787029
GR00302	Leprosy	0.112480573
GR00303	Light vs. dark hair color	0.110772673
GR00304	Longevity	0.005771506
GR00305	Longevity	0.012461137
GR00306	Longevity	0.008818991
GR00307	Lung adenocarcinoma	0.020292166
GR00308	Lung adenocarcinoma	0.017742513
GR00309	Lung adenocarcinoma	0.028713341
GR00310	Lung adenocarcinoma	0.016894924
GR00311	Lung cancer	0.022623669
GR00312	Lung cancer	0.009303033
GR00313	Lung cancer	0.000899019
GR00314	Lung cancer	0.008773378
GR00315	Major depressive disorder	0.021232578
GR00316	Major depressive disorder	0.021918913
GR00317	Major depressive disorder	0.031924004
GR00318	Major depressive disorder	0.008594186
GR00319	Major depressive disorder	0.001796614
GR00320	Major depressive disorder	0.093204755
GR00321	Major depressive disorder	0.113217551
GR00322	Major depressive disorder	0.075236357
GR00323	Major depressive disorder	0.131593953
GR00324	Major mood disorders	0.002518386
GR00325	Male-pattern baldness	0.121084438
GR00326	Marginal zone lymphoma	0.035517708
GR00327	Melanoma	0.001886008
GR00328	Melanoma	0.040532491
GR00329	Meningioma	0.01859283
GR00330	Meningioma	0.006576666
GR00331	Meningococcal disease	0.017267286
GR00332	Mesial temporal lobe epilepsy	0.005423004
GR00333	Mesial temporal lobe epilepsy	0.014282101
GR00334	Migraine	0.073711771
GR00335	Migraine	0.002755316
GR00336	Migraine	0.005754175

GR00337	Migraine	0.033522022
GR00338	Migraine	0.0266128
GR00339	Migraine	0.016704531
GR00340	Migraine	0.042740846
GR00341	Migraine	0.050953244
GR00342	Migraine	0.035697629
GR00343	Migraine	0.016879262
GR00344	Mitral valve prolapse	0.042545002
GR00345	Morning vs. evening chronotype	0.014771931
GR00346	Mortality in heart failure	0.084159468
GR00347	Mortality in heart failure	0.039216859
GR00348	Mortality in heart failure	0.005810295
GR00349	Mucinous ovarian carcinoma	0.037683103
GR00350	Multiple cancers	0.005031245
GR00351	Multiple cancers	0.002052937
GR00352	Multiple cancers	0.004961515
GR00353	Multiple cancers	0.004981674
GR00354	Multiple myeloma	0.01877838
GR00355	Multiple myeloma	0.099548832
GR00356	Multiple myeloma	0.047600503
GR00357	Multiple myeloma	0.027536656
GR00358	Multiple myeloma	0.062994232
GR00359	Multiple myeloma	0.125777425
GR00360	Multiple myeloma	0.152653675
GR00361	Multiple myeloma	0.112659954
GR00362	Multiple myeloma	0.040948437
GR00363	Multiple sclerosis	0.043567172
GR00364	Myeloproliferative neoplasms	0.018935763
GR00365	Myocardial infarction	0.031520528
GR00366	Myopia (pathological)	0.034021276
GR00367	Myopic maculopathy	0.01856891
GR00368	Narcolepsy	0.050030281
GR00369	Narcolepsy	0.046873759
GR00370	Nasopharyngeal carcinoma	0.082867663
GR00371	Neuroblastoma	0.01483877
GR00372	Non-cardia gastric cancer	0.037716691
GR00373	Non-cardia gastric cancer	0.013290882
GR00374	Non-small cell lung cancer	0.022057408

GR00375	Nonsyndromic cleft lip with or without cleft palate	0.075608393
GR00376	Obesity	0.098733769
GR00377	Obesity	0.015721298
GR00378	Ossification of the posterior longitudinal ligament of the spine	0.051716579
GR00379	Osteoarthritis	0.001672743
GR00380	Osteoarthritis	0.000974606
GR00381	Osteoarthritis	0.002494576
GR00382	Osteoarthritis	0.001845007
GR00383	Osteoarthritis	0.004193489
GR00384	Osteoarthritis	0.00195496
GR00385	Osteoarthritis	0.002922793
GR00386	Osteoarthritis	0.018578867
GR00387	Osteoarthritis	0.008021126
GR00388	Osteoarthritis	0.000645973
GR00389	Osteoarthritis	0.000229546
GR00390	Osteoarthritis	0.006433369
GR00391	Osteoarthritis	0.000127462
GR00392	Osteoarthritis	0.010252254
GR00393	Osteoarthritis	0.002428762
GR00394	Osteoporosis	0.007403442
GR00395	Osteoporosis	0.011540439
GR00396	Ovarian cancer	0.016626178
GR00397	Ovarian cancer	0.005203548
GR00398	Ovarian cancer	0.029749261
GR00399	Ovarian cancer	0.015756605
GR00400	Paget's disease of bone	0.11265716
GR00401	Pain	0.003669549
GR00402	Pancreatic cancer	0.0088485
GR00403	Pancreatic cancer	0.020664923
GR00404	Pancreatic cancer	0.004576326
GR00405	Pancreatic cancer	0.019372069
GR00406	Pancreatitis	0.038531639
GR00407	Parkinson's disease	0.023891217
GR00408	Parkinson's disease	0.014151241
GR00409	Parkinson's disease	0.037396297
GR00410	Parkinson's disease	0.02117897
GR00411	Parkinson's disease	0.044928409

GR00412	Parkinson's disease	0.065628889
GR00413	Parkinson's disease	0.064947411
GR00414	Parkinson's disease	0.059609111
GR00415	Pathological myopia	0.011414182
GR00416	Pathological myopia	0.009392818
GR00417	Pathological myopia	0.005383183
GR00418	Periodontitis	0.005733699
GR00419	Peripheral artery disease	0.005950886
GR00420	Plantar fascial disorders	0.000650417
GR00421	Polycystic ovary syndrome	0.049405654
GR00422	Primary biliary cholangitis	0.108521464
GR00423	Primary biliary cholangitis	0.168778978
GR00424	Primary biliary cholangitis	0.044073094
GR00425	Primary biliary cholangitis	0.063903534
GR00426	Primary open-angle glaucoma	0.006113139
GR00427	Primary sclerosing cholangitis	0.016860351
GR00428	Progressive supranuclear palsy	0.260690268
GR00429	Progressive supranuclear palsy	0.00651826
GR00430	Progressive supranuclear palsy	0.017385685
GR00431	Prostate cancer	0.037336022
GR00432	Prostate cancer	0.004880294
GR00433	Prostate cancer	0.002755316
GR00434	Prostate cancer	0.006591922
GR00435	Prostate cancer	0.007694849
GR00436	Prostate cancer	0.012092802
GR00437	Prostate cancer	0.023114387
GR00438	Prostate cancer	0.034007403
GR00439	Prostate cancer	0.141671608
GR00440	Prostate cancer	0.004087186
GR00441	Prostate cancer	0.013263933
GR00442	Prostate cancer	0.125502507
GR00443	Prostate cancer	0.036620931
GR00444	Prostate cancer	0.08792687
GR00445	Prostate cancer	0.011899264
GR00446	Prostate cancer	0.011526175
GR00447	Psoriasis	0.020236227
GR00448	Psoriasis	0.033066348
GR00449	Psoriasis	0.124705641

GR00450	Psoriatic arthritis	0.075310164
GR00451	Pulmonary artery enlargement	0.017126015
GR00452	Pulmonary artery enlargement	0.019359247
GR00453	Red vs non-red hair color	0.312843634
GR00454	Red vs. non-red hair color	0.02780307
GR00455	Renal cell carcinoma	0.010859326
GR00456	Renal cell carcinoma	0.010229543
GR00457	Renal gout	0.111210121
GR00458	Renal gout	0.143977121
GR00459	Response to Dalcetrapib treatment in acute coronary syndrome	0.027286116
GR00460	Response to metformin	0.013311215
GR00461	Restless legs syndrome	0.080015375
GR00462	Restless legs syndrome	0.050447072
GR00463	Rhegmatogenous retinal detachment	0.014978066
GR00464	Rheumatoid arthritis	0.005328866
GR00465	Rheumatoid arthritis	0.003042565
GR00466	Rheumatoid arthritis	0.055045324
GR00467	Rheumatoid arthritis	0.055469965
GR00468	Sarcoidosis	0.003822456
GR00469	Sarcoidosis	0.016984883
GR00470	Sasang constitutional medicine type (So-Eum)	0.007287019
GR00471	Schizophrenia	0.011696159
GR00472	Schizophrenia	0.053531251
GR00473	Schizophrenia	0.040260912
GR00474	Sclerosing cholangitis and ulcerative colitis (combined)	0.019663288
GR00475	Scoliosis	0.007895798
GR00476	Scoliosis	0.028621338
GR00477	Scoliosis	0.008440551
GR00478	Shingles	0.004464138
GR00479	Sjogren's syndrome	0.13009326
GR00480	Skin sensitivity to sun	0.02780307
GR00481	Smoking behavior	0.001048274
GR00482	Smoking behavior	0.000342313
GR00483	Smoking behavior	0.009165292
GR00484	Sporadic neuroblastoma	0.080754199
GR00485	Sporadic pituitary adenoma	0.017342659
GR00486	Squamous cell carcinoma	0.007006683

GR00487	Squamous cell carcinoma	0.034586255
GR00488	Stroke	0.006030095
GR00489	Stroke	0.013995513
GR00490	Stroke	0.002033565
GR00491	Stroke	0.003338235
GR00492	Stroke	0.00461742
GR00493	Stroke	0.008782398
GR00494	Stroke	0.010304228
GR00495	Stroke	0.006084844
GR00496	Sudden cardiac arrest	0.003558252
GR00497	Supraventricular ectopy	0.026160487
GR00498	Survival in breast cancer	0.034824375
GR00499	Survival in colorectal cancer	0.002909558
GR00500	Survival in colorectal cancer	0.004571026
GR00501	Survival in colorectal cancer	0.021119802
GR00502	Susceptibility to persistent hepatitis B virus infection	0.007381044
GR00503	Systemic lupus erythematosus	0.005541145
GR00504	Systemic lupus erythematosus	0.009112039
GR00505	Systemic lupus erythematosus	0.179247159
GR00506	Systemic lupus erythematosus	0.353421906
GR00507	Systemic lupus erythematosus	0.017106523
GR00508	Systemic sclerosis	0.064608715
GR00509	Systemic sclerosis	0.024335964
GR00510	Systemic sclerosis	0.124107232
GR00511	Systemic sclerosis	0.181583325
GR00512	Systemic sclerosis	0.006159744
GR00513	Systemic sclerosis	0.012824084
GR00514	Taxane-induced peripheral neuropathy in breast cancer	0.181310486
GR00515	Testicular germ cell cancer	0.123528788
GR00516	Testicular germ cell cancer	0.162714565
GR00517	Testicular germ cell cancer	0.024678861
GR00518	Testicular germ cell cancer	0.016253323
GR00519	Thyroid cancer	0.030497438
GR00520	Thyroid cancer	0.078088926
GR00521	Thyroid cancer	0.011690141
GR00522	Tuberculosis	0.063991801
GR00523	Tuberculosis	0.003940065

GR00524	Type 1 diabetes	0.021320715
GR00525	Type 1 diabetes	0.051835334
GR00526	Type 1 diabetes	0.005037086
GR00527	Type 1 diabetes nephropathy	0.00915456
GR00528	Type 1 diabetes nephropathy	0.001504595
GR00529	Type 1 diabetes nephropathy	0.046037617
GR00530	Type 2 diabetes	0.044935806
GR00531	Type 2 diabetes	0.001105361
GR00532	Type 2 diabetes	0.022416555
GR00533	Type 2 diabetes	0.014658754
GR00534	Type 2 diabetes	0.054303551
GR00535	Type 2 diabetes	0.016754351
GR00536	Type 2 diabetes	0.059338112
GR00537	Type 2 diabetes	0.027245553
GR00538	Type 2 diabetes	0.083002864
GR00539	Type 2 diabetes	0.006839448
GR00540	Type 2 diabetes	0.004361046
GR00541	Type 2 diabetes	0.001610264
GR00542	Type 2 diabetes	0.008502074
GR00543	Type 2 diabetes	0.0078655
GR00544	Type 2 diabetes	0.005614025
GR00545	Type 2 diabetes	0.023680631
GR00546	Type 2 diabetes	0.023838184
GR00547	Type 2 diabetes	0.001134864
GR00548	Type 2 diabetes	0.03444121
GR00549	Type 2 diabetes	0.006568327
GR00550	Type 2 diabetes	0.017650835
GR00551	Type 2 diabetes	0.094405465
GR00552	Type 2 diabetes	0.041647764
GR00553	Ulcerative colitis	0.05540542
GR00554	Ulcerative colitis	0.10520555
GR00555	Ulcerative colitis	0.061004829
GR00556	Ulcerative colitis	0.054964378
GR00557	Upper aerodigestive tract cancers	0.01048981
GR00558	Urinary bladder cancer	0.009484496
GR00559	Urinary bladder cancer	0.013220862
GR00560	Uterine fibroids	0.023781525
GR00561	Uterine fibroids	0.015926844

GR00562	Venous thromboembolism	0.143738499
GR00563	Venous thromboembolism	0.067389334
GR00564	Venous thromboembolism	0.082459649
GR00565	Viral capsid antigen IgG seropositivity	0.003676288
GR00566	Vitiligo	0.009634001
GR00567	Vitiligo	0.032379763
GR00568	Vitiligo	0.11318408
GR00569	Wilms tumor	0.046496324
