# Supplementary material for: Assessing the performance of genome-wide association studies for predicting disease risk
Source: PLoS One. 2019 Dec 5;14(12):e0220215. doi: 10.1371/journal.pone.0220215 (PMC6894795; doi:10.1371/journal.pone.0220215)
Supplement: S2 Table — (PDF) [file pone.0220215.s002.pdf]

S2 Table

Study Purpose	AUROC	PMID
Crohn's Disease	0.75	28851283
Alzheimer's Disease	0.68	28727176
Alzheimer's Disease	0.67	26490334
Skin Colour	0.73-0.97	28500464
Pulmonary tuberculosis	0.64	28355295
Breast Cancer	0.64	28205043
Obesity	0.57	23701538
Esophageal Squamous-cell Carcinoma	0.63	23536576
Common Psoriasis	0.76	28537254
Osteoporotic Fracture	0.55	23572424
10 different conditions	0.6-0.8	28065900
Renal Cell Carcinoma	0.66	27229762
Alcohol Dependence	0.55	23362995
Chronic Myelogenous Leukemia	0.61	26474455
Venous Thromboembolism	0.66	25472531
Alzheimer's Disease	0.64	26543236
Leprosy	0.71	30231057
Colorectal Cancer	0.59	29908285
Head Hair Shape	0.664-0.789	30268682
Celiac Disease	0.86-0.9	24550740
Non-alcoholic Steatohepatitis	0.65	29385134
Type 2 Diabetes	0.64	24947790
Prostate Cancer	0.60	27140652
Crohn's Disease	0.78	27076762
Lung Cancer	0.55	23228068
Triple-Negative Breast Cancer	0.94	28918577
Psoriasis	0.78	28617847
Obesity	0.73	23629956
Trichloroethylene induced hypersensitivity syndrome	0.81	26190474
Bipolar Disorder	0.60	26178159
Obesity in people with major depressive disorder	0.58	25903154
Axial Spondyloarthritis	0.83	27749235
Crohn's Disease	0.73-0.97	28052082
Straight Hair	0.69	26414620

Nasopharyngeal Carcinoma	0.52	25180181
IVF embryo transfer implantation failures	0.75	28388872
Cutaneous Melanoma	0.64	30060076
Graves' Disease	0.70	30649410
Age Related Macular Degeneration	0.77	24576882
Breast Cancer	0.63	30554720
Osteoporosis	0.67	28580384
Type 2 Diabetes	0.86	29099854
Prostate Biopsy	0.61	24265090
Breast Cancer	0.57	18612136
Type 2 Diabetes	0.64	23956346
Multiple Sclerosis	0.69	22164203
Breast Cancer	0.59	29302764
Non-small Cell Lung Cancer	0.50	23720679
Non-melanoma Skin Cancer	0.66	30085400
Ulcerative Colitis	0.86	24241240
Alzheimer's Disease	0.70	26086184
Rheumatoid Arthritis	0.79	20032229
Rheumatoid Arthritis	0.66	24068971
Prostate Cancer	0.60	26431041
Prostate Biopsy	0.59	22652152
Metabolic Syndrome	0.64	24198294
Breast Cancer	0.65	22269215
Breast Cancer	0.74	23354978
Alzheimer's Disease	0.69	29784544
Effect of Immunosuppressive Treatment in First Kidney Transplant	0.69	27777962
Myocardial Infarcation	0.60	29340220
Rheumatoid Arthritis	0.59	21980439
Non-alcoholic Steatohepatitis	0.56	25597287
Eye color	0.6-0.889	27221533
Breast Cancer	0.59	25380502
Predictors of treatment nonresponse to the first anti-TNF inhibitor in ankylosing spondylitis	0.77	24337767
Hair Color	0.66-0.86	21197618
Breast Cancer	0.68	21212067
Type 2 Diabetes	0.69	30767168
Breast Cancer	0.58	22314178
Alzheimer's Disease	0.74	25720397

Type 1 Diabetes	0.92	30655379
Systemic Lupus Erythematosus	0.71	29967481
Prostate Cancer	0.66	23071574
Psoriasis	0.72	21559375
Coronary Artery Disease	0.6-0.8	20729558
Breast Cancer	0.60	22585702
Major Depressive Disorder	<0.54	25279001
Type 2 Diabetes Mellitus	0.62	20384434
Oral Malignancy	0.61	30657779
Rheumatoid Arthritis	0.70	20309765
Metastic Colorectal Cancer	0.88	25372392
Elevated Liver Fat Content	0.66	23804528
Chronic Hepatitis C Therapy	0.75	23615070
Systemic Lupus Erythematosus	0.76	26689915
Type 1 Diabetes	0.79	19956648
Relapse in recipients of allogeneic haematopoietic stem cell transplantation	0.72	30089915
Breast Cancer	0.65	27279675
Carotid Atherosclerosis	0.65	20941391
Type 2 Diabetes	0.60	18694974
Glaucoma	0.62-0.94	30352225
Type 2 Diabetes	0.64	19197355
Type 2 Diabetes	0.63	20571754
Breast cancer	0.64	27565998
Prostate Cancer	0.66	28827750
Colorectal Cancer	0.56	28233817
Stroke Risk	0.65	30073812
Oral Cancer Recurrence	0.64	28400480
Breast Cancer	0.58	22972951
Multiple Sclerosis	0.59	23903824
Type 1 Diabetes	0.88	26577414
Type 2 Diabetes	0.58	17020404
Type 2 Diabetes	0.60	18591388
Type 2 Diabetes	0.63	19247372
Type 2 Diabetes	0.60	19404609
Type 2 Diabetes	0.62	19862325
Colorectal Cancer	0.57	22490517
Age Related Macular Degeneration	0.82	22666427
Crohn's Disease	0.71	21548950

Prostate Cancer	0.61	20620408
Age Related Macular Degeneration	0.73	18596911
Age Related Macular Degeneration	0.77	19825847
