# Supplementary material for: Assessing the performance of genome-wide association studies for predicting disease risk
Source: PLoS One. 2019 Dec 5;14(12):e0220215. doi: 10.1371/journal.pone.0220215 (PMC6894795; doi:10.1371/journal.pone.0220215)
Supplement: S3 Table — (PDF) [file pone.0220215.s003.pdf]

S3 Table

Number of SNPs	Number of Studies in GWAS Central
1	165
2	78
3	64
4	54
5	37
6	31
7	19
8	21
9	15
10	20
11	3
12	4
13	4
14	7
15	3
16	4
17	4
18	1
19	1
20	2
21	2
23	4
25	1
26	1
27	1
28	1
29	3
30	3
32	2
34	2
36	2
37	1
38	1
40	1
44	1

50	6
