# Supplementary material for: Assessing the performance of genome-wide association studies for predicting disease risk
Source: PLoS One. 2019 Dec 5;14(12):e0220215. doi: 10.1371/journal.pone.0220215 (PMC6894795; doi:10.1371/journal.pone.0220215)
Supplement: S4 Table — (PDF) [file pone.0220215.s004.pdf]

S4 Table

Phenotype/Condition	AUROC
Black vs. non-black hair color	0.97
Celiac disease	0.84
Dupuytren's disease	0.75
Age-related macular degeneration	0.75
Progressive supranuclear palsy	0.71
Heart failure	0.66
Testicular germ cell cancer	0.65
Inflammatory bowel disease	0.64
Autoimmune hepatitis type-1	0.63
Chronic hepatitis B infection	0.63
Brugada syndrome	0.63
Blond vs. brown hair color	0.63
Atrial fibrillation	0.62
Disease-free survival in breast cancer	0.62
Taxane-induced peripheral neuropathy in breast cancer	0.62
Crohn's disease	0.62
Systemic lupus erythematosus	0.61
Craniofacial microsomia	0.61
Paget's disease of bone	0.61
Leprosy	0.61
Hypertriglyceridemia	0.61
Nasopharyngeal carcinoma	0.61
Anti-dsDNA status	0.61
Interstitial lung disease	0.60
Male-pattern baldness	0.60
Late onset Alzheimer's disease	0.60
Chronic lymphocytic leukemia	0.60
Hepatitis B	0.60
Ulcerative colitis	0.60
Venous thromboembolism	0.59
Graves disease	0.59
Psoriasis	0.59
Sjogren's syndrome	0.59
Hodgkin's lymphoma	0.59
Infantile hypertrophic pyloric stenosis	0.59

Primary biliary cholangitis	0.59
Restless legs syndrome	0.58
Insomnia (caffeine-induced)	0.58
Renal gout	0.58
Burning and freckling	0.58
High serum lipase activity	0.58
Prostate cancer	0.57
Kawasaki disease	0.57
Idiopathic pulmonary fibrosis	0.57
Dementia with Lewy bodies	0.57
Febrile seizures	0.57
Goiter	0.57
Sporadic neuroblastoma	0.57
Gout	0.57
Polycystic ovary syndrome	0.57
Early-onset obesity	0.56
Multiple sclerosis	0.56
Corneal astigmatism	0.56
Psoriatic arthritis	0.56
Immunoglobulin light chain (AL) amyloidosis	0.56
Nonsyndromic cleft lip with or without cleft palate	0.56
Blond vs non-blond hair color	0.56
Supraventricular ectopy	0.56
Major depressive disorder	0.56
Ewing sarcoma	0.56
Glioma	0.56
Acute-on-chronic liver failure in hepatitis B	0.56
Bronchopulmonary dysplasia	0.56
Pancreatitis	0.56
Myocardial infarction	0.56
Migraine	0.56
End-stage renal disease in Type 1 diabetics (Women)	0.56
Case-only systemic lupus erythematosus	0.56
Thyroid cancer	0.56
Hepatitis C induced liver cirrhosis	0.55
Wilms tumor	0.55
Gallbladder cancer	0.55
Light vs. dark hair color	0.55
Follicular lymphoma	0.55

Aggressive periodontitis	0.55
Acute lymphoblastic leukemia (childhood)	0.55
Survival in breast cancer	0.55
Adolescent idiopathic scoliosis	0.55
Multiple myeloma	0.55
Myeloproliferative neoplasms	0.55
Freckles	0.55
Brown vs. non-brown hair color	0.55
Lung adenocarcinoma	0.55
Ossification of the posterior longitudinal ligament of the spine	0.55
Systemic sclerosis	0.55
Primary sclerosing cholangitis	0.54
Parkinson's disease	0.54
Creutzfeldt-Jakob disease	0.54
Obesity	0.54
Response to Dalcetrapib treatment in acute coronary syndrome	0.54
Leishmaniasis (visceral)	0.54
Narcolepsy	0.54
Marginal zone lymphoma	0.54
Melanoma	0.54
Rheumatoid arthritis	0.54
Vitiligo	0.54
Diffuse large B cell lymphoma	0.54
Myopic maculopathy	0.54
Acne (severe)	0.54
Non-small cell lung cancer	0.54
Helicobacter pylori serologic status	0.54
Epithelial ovarian cancer	0.54
Mucinous ovarian carcinoma	0.54
Abdominal aortic aneurysm	0.54
Myopia (pathological)	0.54
Allergic sensitization	0.54
Ovarian cancer	0.54
Red vs. non-red hair color	0.54
Skin sensitivity to sun	0.54
B cell non-Hodgkin lymphoma	0.54
Amyotrophic lateral sclerosis	0.54
Mortality in heart failure	0.54

Cholangiocarcinoma in primary sclerosing cholangitis	0.54
Meningioma	0.54
Aortic valve stenosis	0.53
Cleft lip	0.53
Allergic disease	0.53
Endometrial cancer	0.53
Anorexia nervosa	0.53
Diabetic nephropathy	0.53
Response to metformin	0.53
Neuroblastoma	0.53
Periodontitis	0.53
Fractures (vertebral)	0.53
Mitral valve prolapse	0.53
Scoliosis	0.53
Mesial temporal lobe epilepsy	0.53
Chronic bronchitis in chronic obstructive pulmonary disease	0.53
Susceptibility to persistent hepatitis B virus infection	0.53
Digestive system disease	0.53
Early onset inflammatory bowel disease	0.53
Chronic hepatitis C infection	0.53
Autism	0.53
Gastric cancer	0.53
Sporadic pituitary adenoma	0.53
Irritable bowel syndrome	0.53
Calcific aortic valve stenosis	0.53
Longevity	0.52
Coronary artery disease	0.52
Sclerosing cholangitis and ulcerative colitis (combined)	0.52
Cardiac repolarization	0.52
Type 2 diabetes	0.52
Breast cancer	0.52
Pulmonary artery enlargement	0.52
Epilepsy	0.52
Alzheimer's disease	0.52
Behcet's disease	0.52
Knee osteoarthritis	0.52
Urinary bladder cancer	0.52
Type 1 diabetes	0.52
Rhegmatogenous retinal detachment	0.52

Non-cardia gastric cancer	0.52
Bipolar disorder	0.52
Hepatocellular carcinoma	0.52
Intracranial aneurysm	0.52
Squamous cell carcinoma	0.52
Primary open-angle glaucoma	0.52
Morning vs. evening chronotype	0.52
Cardia gastric cancer	0.52
Congenital heart malformation	0.52
Pancreatic cancer	0.52
Inguinal hernia	0.52
Osteoporosis	0.52
Hypertension	0.52
Esophageal cancer	0.52
Endometriosis	0.52
Uterine fibroids	0.52
Disc degeneration (lumbar)	0.52
Chronic obstructive pulmonary disease	0.52
Cervical cancer	0.52
Combined Crohn's disease and sarcoidosis	0.52
Peripheral artery disease	0.52
Sarcoidosis	0.52
Diarrhoeal Disease	0.52
Anxiety disorder	0.52
Intracerebral hemorrhage	0.52
Atopic dermatitis	0.52
Glaucoma	0.52
Hyperemesis gravidarum	0.52
Survival in colorectal cancer	0.52
Type 1 diabetes nephropathy	0.52
Pain	0.51
Basal cell carcinoma	0.51
Upper aerodigestive tract cancers	0.51
Tuberculosis	0.51
Renal cell carcinoma	0.51
Curve progression in adolescent idiopathic scoliosis	0.51
Sudden cardiac arrest	0.51
Drinking behavior	0.51
Blue vs. green eyes	0.51

Major mood disorders	0.51
Barrett's esophagus	0.51
Drug abuse	0.51
Asthma	0.51
Pathological myopia	0.51
Cardiovascular disease risk factor	0.51
Arthritis (juvenile idiopathic)	0.51
Multiple cancers	0.51
Smoking behavior	0.51
Lung cancer	0.51
Schizophrenia	0.51
Meningococcal disease	0.51
Red vs non-red hair color	0.51
Epstein Barr virus nuclear antigen 1 IgG seropositivity	0.50
Viral capsid antigen IgG seropositivity	0.50
Sasang constitutional medicine type (So-Eum)	0.50
Kidney disease	0.50
Kidney stones	0.50
Plantar fascial disorders	0.50
Shingles	0.50
Colon cancer	0.50
Carotid intima media thickness, plaque	0.50
Enteric fever	0.50
Colorectal cancer	0.50
Chronic kidney disease	0.50
Bladder cancer	0.50
Ischemic stroke	0.50
Stroke	0.49
Osteoarthritis	0.47
