# Supplementary material for: Assessing the performance of genome-wide association studies for predicting disease risk
Source: PLoS One. 2019 Dec 5;14(12):e0220215. doi: 10.1371/journal.pone.0220215 (PMC6894795; doi:10.1371/journal.pone.0220215)
Supplement: Attachment — Submitted filename: Response-letter-PLOS ONE - DSW.docx [file pone.0220215.s005.docx]

Sept. 29, 2019

Dear Dr. Devaney,

Please find attached our revised manuscript (PONE-D-19-19050) entitled: “Assessing the performance of genome-wide association studies for predicting disease risk” by Jonas Patron, Arnau Serra-Cayuela, Beomsoo Han, Carin Li and myself. We wish to thank the reviewer for their comments and suggestions. We have tried our best to address all the reviewer’s comments by modifying the paper and have provided detailed responses or described our modifications to the manuscript in the attached pages. We have also produced both a clean version and marked-up version of the manuscript with all changes indicated in red so that the edits or modifications can be more easily seen.

For Reviewer #1:

1. On page 7&8, it seems authors first simulate the SNP profile and then assign the healthy/disease statuses. Shouldn’t it simulate the SNP profile given the healthy/disease statuses? A detailed explanation is needed for this part.

   **Response:** We appreciate the reviewer’s concerns and apologize for the confusion. Indeed, we simulate the SNP profiles given the healthy/disease status of the cohort. Note from equations (1)-(4) on pg. 7&8 we start by determining or inputting the number of cases and controls. That is, we always start by knowing the healthy/disease status of the individuals in the cohort, we just don’t know the SNP profile of said individuals. Next, by knowing the risk allele frequency in the controls and the odds ratio between the cases and controls we calculate the risk allele frequency in the cases. Once we know the risk allele frequency in both the case and control groups, we appropriately assign the SNP profiles to each group. We have added portions of this text to the manuscript to explain the process in more detail.
2. On page 10, what is the variance inflation factor? How was it used to determine if there is multicollinearity?

   **Response:** Given a regression model with multiple variables, the variance inflation factor is the quotient of the variance from a model which regresses one of the predictor variables against all the others. Multicollinearity was determined to exist when at least two variables showed inflated coefficients, as quantified by the calculation of the variance inflation factor being equal to infinity. We also tried other variance inflation factor cutoff values, however because the differences in the AUC estimates were so small (<0.009) and because standard logistic regression is more easily interpretable than its ridge regression counterpart, we found it appropriate to restrict the use of ridge regression only to models with extreme (i.e. divergent) variance inflation factor estimates. We have added portions of this text to the manuscript to explain this concept in more detail.
3. On page 14, what are odd/even SNPs?

   **Response:** The characterization of SNPs as odd or even is simply referring to their position in a list—those found at odd numbered positions in the list are “odd SNPs” and those that are found in even numbered positions are called “even” SNPs. It is a simple scheme to split the SNPs in an approximately 50-50 fashion. Consider Table R1 (see below), which is a table with SNPs. The 1^st^, 3^rd^, and 5^th^ SNPs would be the odd SNPs and the 2^nd^ and 4^th^ SNPs would be the even SNPs.

   Table R1

| Accession | Control size | Case size | Risk allele freq | OR | Odd/Even |
| --- | --- | --- | --- | --- | --- |
| rs7903146 | 2598 | 2309 | 0.3 | 1.36 | odd |
| rs5219 | 2598 | 2309 | 0.36 | 1.25 | even |
| rs10811661 | 2598 | 2309 | 0.85 | 1.21 | odd |
| rs1801282 | 2598 | 2309 | 0.87 | 1.21 | even |
| rs2641348 | 2598 | 2309 | 0.11 | 1.15 | odd |

4. On page 15, authors claimed that “This was important because the SNP inheritance model in published GWA studies may vary from SNP to SNP, and is not typically disclosed. Thus, for each of the 7 WTCCC disease studies we calculated the true AUROC under these four SNP inheritance schemes.” I do not see the point to have four schemes using logistic and ridge regression. The scheme is automatically handled when the model is being fitted. Why do we need to restrict the inheritance scheme explicitly?

   **Response:** This is an excellent point. What were doing was conducting two different and independent modeling steps. In the first step, we were trying to calculate the true AUROC of a given WTCCC disease study. To do this we had to convert the raw GWAS data into dummy variables to which we could apply logistic regression. Take Table R2 (below) for example. In this example, there is one individual and we have information about four of his/her SNPs. To run a regression analysis the information in columns “1^st^ Allele” and “2^nd^ Allele” must be converted to a single number (either a 0 or 1) known as the dummy variable. Then, in the second step we would run our logistic/ridge regression to estimate the AUROC (in this step the choice between logistic or ridge regression would be handled automatically). Note that in this simple example a change in the choice of the SNP inheritance scheme produces three distinct results: (1,1,0,1), (0,1,0,0), and (1,1,0,0). As a result, when we run our standard regression techniques we could expect to produce 3 different AUROCs, all of which can be interpreted as the “true” AUROC.

Table R2

| SNP | Risk Allele | 1^st^ Allele | 2^nd^ Allele | Dominant Inheritance | Recessive Inheritance | Dominant (odd) – Recessive (even) |
| --- | --- | --- | --- | --- | --- | --- |
| #1 | A | T | A | 1 | 0 | 1 |
| #2 | A | A | A | 1 | 1 | 1 |
| #3 | T | A | A | 0 | 0 | 0 |
| #4 | C | C | G | 1 | 0 | 0 |

As such, to rigorously test GWIZ’s AUROC predictions, we had to ensure that we could accurately predict the AUROC which resulted from any one of these inheritance schemes. In our test set we could restrict the inheritance scheme explicitly, however in practice, given a set of odds ratios we cannot necessarily know which inheritance scheme produced them.

5. In Table 2, some AUROCs are close to 0.5. Does it mean the report SNPs has little or no contribution to the classification accuracy? Do we have any explanation that low p-value SNPs have little discrimination power?

   **Response:** With regard to the first question, yes, when the AUROCs are close to 0.5 it does indicate that the reported SNPs has/have little or no contribution to the classification accuracy. With regard to the second question, the size of the p-value and its influence on the AUROC is primarily affected by the associated SNP’s odds ratio and allele frequency. In other words, low p-values can arise from statistically significant effects of small magnitude. In GWA studies, the ‘effect’ we are looking for is that of differences in allele frequencies between case and control groups. And the magnitude of the effect in question is measured by the odds ratios. A SNP with a p-value of 10^-80^ but with an odds ratio close to 1 (which is typical of most GWAS SNPs) will have a very small effect on the AUROC. A low odds ratio often arises from a very low SNP/allele frequency. A SNP with an odds ratio of 3 but a p-value of just 10^-10^ will have a large effect on the AUROC.
6. On page 18, there is a comparison of AUROCs between GWAS-derived vs. non-GWAS-derived risk factors. How were those AUROCs of non-GWAS-derived risk factors calculated? Did they use the same logistic or ridge regression models? If not, the comparison may not be fair. The difference may be caused by the models not the risk factors.

   **Response:** The AUROCs of the non-GWAS derived risk factors were calculated using a mixture of different approaches. About 1/3 of the values quoted in this table used logistic regression while the others used either PLS-DA or random forest methods. It is true that the choice of the classification model can affect the performance and the reported AUROC but generally most biostatisticians will test multiple classification models (logistic regression, PLS-DA, random forest, SVM regression, etc.) and will quote the best performing model. Our own experience in using many different classifiers for many other related projects is that the AUROC differences between classifiers are generally quite small (<5%) although rare exceptions do occur. Overall, we believe the AUROC differences accurately reflect the risk factors and that they are not a (significant) function of the choice of classifier. We have added this information to the manuscript.
7. Will the authors release the developed tool as open source? The impact will be limited if the research community cannot verify and apply the computational method.

   **Response:** The code is freely available for download at https://bitbucket.org/wishartlab/gwiz-rscript/src/master/, and is compatible with a wide variety of UNIX platforms, Mac OS and Windows operating systems. The above link to bitbucket is cited in the original manuscript on page 11, however to reduce confusion in the future we have also mentioned this link in the abstract.

In addition to these edits we have also made some small changes to the manuscript by adding a few missing references and elaborating on GWIZ’s comparison to other programs. This led to a small update to Figure 3. Once again, we would like to thank the reviewer for their comments and suggestions. We hope that the explanations we have provided are satisfactory and certainly the changes they suggested have significantly improved the overall clarity and quality of the manuscript. We hope these edits are acceptable and are looking forward to seeing the manuscript published in PLOS ONE soon.

Sincerely,

David Wishart
